# Bile Acids Induce Alterations in Mitochondrial Function in Skeletal Muscle Fibers

**DOI:** 10.3390/antiox11091706

**Published:** 2022-08-30

**Authors:** Johanna Abrigo, Hugo Olguín, Danae Gutierrez, Franco Tacchi, Marco Arrese, Daniel Cabrera, Mayalen Valero-Breton, Alvaro A. Elorza, Felipe Simon, Claudio Cabello-Verrugio

**Affiliations:** 1 Laboratory of Muscle Pathology, Fragility and Aging, Faculty of Life Sciences, Universidad Andres Bello, Santiago 8370146, Chile; 2 Millennium Institute on Immunology and Immunotherapy, Faculty of Life Sciences, Universidad Andres Bello, Santiago 8370146, Chile; 3 Center for the Development of Nanoscience and Nanotechnology (CEDENNA), Universidad de Santiago de Chile, Santiago 8350709, Chile; 4 Laboratory of Tissue Repair and Adult Stem Cells, Department of Cellular and Molecular Biology, Faculty of Biological Sciences, Pontificia Universidad Católica de Chile, Santiago 8330077, Chile; 5 Departamento de Gastroenterología, Escuela de Medicina. Centro de Envejecimiento y Regeneración (CARE), Facultad de Ciencias Biológicas, Pontificia Universidad Católica de Chile, Santiago 8330077, Chile; 6 Facultad de Ciencias Médicas, Universidad Bernardo O Higgins, Santiago 8370993, Chile; 7 Institute of Biomedical Sciences, Faculty of Medicine and Life Sciences, Universidad Andres Bello, Santiago 8370146, Chile; 8 Millennium Nucleus of Ion Channel-Associated Diseases (MiNICAD), Universidad de Chile, Santiago 8370146, Chile; 9 Laboratory of Integrative Physiopathology, Faculty of Life Sciences, Universidad Andres Bello, Santiago 8370146, Chile

**Keywords:** bile acids, mitochondria, sarcopenia, muscle wasting, TGR5 receptor

## Abstract

Cholestatic chronic liver disease is characterized by developing sarcopenia and elevated serum levels of bile acids. Sarcopenia is a skeletal muscle disorder with the hallmarks of muscle weakness, muscle mass loss, and muscle strength decline. Our previous report demonstrated that deoxycholic acid (DCA) and cholic acid (CA), through the membrane receptor TGR5, induce a sarcopenia-like phenotype in myotubes and muscle fibers. The present study aimed to evaluate the impact of DCA and CA on mitochondrial mass and function in muscle fibers and the role of the TGR5 receptor. To this end, muscle fibers obtained from wild-type and TGR5^−/−^ mice were incubated with DCA and CA. Our results indicated that DCA and CA decreased mitochondrial mass, DNA, and potential in a TGR5-dependent fashion. Furthermore, with TGR5 participation, DCA and CA also reduced the oxygen consumption rate and complexes I and II from the mitochondrial electron transport chain. In addition, DCA and CA generated more mitochondrial reactive oxygen species than the control, which were abolished in TGR5^−/−^ mice muscle fibers. Our results indicate that DCA and CA induce mitochondrial dysfunction in muscle fibers through a TGR5-dependent mechanism.

## 1. Introduction

Sarcopenia is classically associated with muscle wasting from aging. However, this concept has changed, and the new definition of sarcopenia indicates that it can also be produced by other causes, such as chronic diseases. Thus, sarcopenia from aging is classified as primary, whereas that produced by chronic illness is considered secondary [1]. Several mechanisms can be involved in the generation and development of sarcopenia, such as unbalanced protein metabolism, oxidative stress, autophagy dysregulation, myonuclear apoptosis, and mitochondrial dysfunction [2]. Mitochondria act as the primary source of ATP for muscle fibers through the normal function of oxidative phosphorylation (OXPHOS). Mitochondrial dysfunction has been described as a critical process in muscle wasting and is characterized by mitochondrial alterations including morphological changes, decreased biomass, and modified OXPHOS components, such as complexes of the electron transport chain (ETC), as well as altered membrane potential and increased reactive oxygen species (ROS) formation [3,4,5].

One of the clinical conditions contributing to the development of sarcopenia is chronic liver disease (CLD), characterized by muscle weakness and atrophy. Several mediators, such as myostatin and hyperammonemia, have been described as participating in the muscle wasting associated with CLD [6]. We have previously demonstrated that a model of cholestatic liver disease led to muscle wasting, evidenced by decreased muscle strength and atrophy, associated with increased levels of plasma bile acids [7,8,9]. In addition, we demonstrated that cholic acid (CA) and deoxycholic acid (DCA) induced a sarcopenia-like phenotype in myotubes and isolated muscle fibers [10]. The effects observed in our studies in vitro and in vivo depended on Takeda G protein-coupled receptor 5 (TGR5) expression, the unique membrane receptor for bile acids found in skeletal muscle.

Various studies have demonstrated that increased concentrations of bile acids have a toxic cellular effect because they can modulate mitochondrial function at different levels. In isolated mitochondria from the liver, it was observed that different bile acids, including DCA, induce dose-dependent membrane depolarization and stimulation of the mitochondrial permeability transition pore [11,12], similar to the effects observed in the mitochondria of cardiomyocytes [13,14]. In brown adipose tissue, the impact of bile acids, such as CA, was associated with an increase in energy expenditure mediated by the activation of thyroid hormone, which induces increased oxygen consumption through the TGR5 receptor [15]. Watanabe et al. (2006) suggested that the response to bile acids from mitochondria in skeletal muscle could be similar to adipose tissue due to their common characteristics [15].

Based on this evidence, we hypothesized that CA and DCA can induce mitochondrial dysfunction through the TGR5 receptor in muscle fibers characterized by altered mitochondrial biomass, mitochondrial potential, oxygen consumption rate, and OXPHOS complex levels, among other properties.

## 2. Materials and Methods

### 2.1. Culture of Isolated Muscle Fibers

C57BL/6J WT male mice (16 weeks old) and C57BL/6J Gpbar1^−/−^ mice (referred to in this study as TGR5^−/−^ mice; 16 weeks old) were anesthetized and euthanized to obtain extensor digitorum longus (EDL) muscles. Muscles were dissected and incubated in an F12 medium supplemented with collagenase I (750 U/mL; Worthington Biochemical Corporation, Lakewood, NJ, USA) and gently stirred for 90 min at 37 °C. The disaggregated muscle was incubated with F12 medium supplemented with 15% horse serum (HS). Furthermore, live fibers were seeded with F12-15% HS medium in a plate covered with Matrigel Matrix GFR (Corning, Glendale, AZ, USA), which was diluted 1:5 in F12 and maintained at 37 °C under an atmosphere of 5% CO_2_ [10,16]. When fibers successfully adhered to the plate, treatments were performed. For the bile acid treatment, cultured fibers were incubated without bile acids (control) or with either 120 µM deoxycholic acid (DCA; Sigma-Aldrich, St. Louis, MO, USA) or 500 µM cholic acid (CA; Sigma-Aldrich, St. Louis, MO, USA) for the times indicated in each figure. All procedures with mice were performed following all applicable international, national, and institutional guidelines for the care and use of animals. Our study obtained the formal approval of the Animal Ethics Committee at the Universidad Andrés Bello (approval number 007/2016). TGR5^−/−^ mice were donated by Dr. Auwerx (Laboratory of Integrative and Systems Physiology, École Polytechnique Fédérale de Lausanne, Switzerland) to Dr. Marco Arrese.

### 2.2. Western Blot

The muscle fibers were solubilized with radio-immunoprecipitation assay (RIPA) buffer (50 mM Tris-HCl, pH 7.4, 150 mM NaCl, 1 mM Na_2_EDTA, 1 mM EGTA, 1% NP-40, 1% sodium deoxycholate) including protease inhibitors (1 mM phenylmethylsulfonyl fluoride, 1 mM cocktail of protease inhibitors) and scraped from the plates to prepare cell extracts. The protein concentrations were determined using a micro-BCA assay. Protein extracts from muscle fibers incubated with DCA, CA, or vehicle were obtained, mixed with loading buffer, and heated to 50 °C for 5 min. Furthermore, the samples were subjected to 12% SDS-PAGE. Electrophoretic transference was applied to polyvinylidene difluoride (PVDF) membranes (Thermo Fisher Scientific, Waltham, MA, USA). The membranes were blocked with 5% skim milk–Tris buffer saline (TBS) and incubated separately with anti-OXPHOS (1:1000; Abcam, Cambridge, MA, USA) and anti-β-actin (1:1000; Abcam, Cambridge, MA, USA) antibodies. The chemiluminescent signals (Thermo Scientific, Waltham, MA, USA) were detected using secondary antibodies and peroxidase-coupled IgG (1:1000) with Fotodyne (Fisher Scientific, St. Waltham, MA, USA). The blots were then quantified by densitometry using ImageJ software (NIH, Bethesda, MD, USA) [17].

### 2.3. Mitochondrial DNA Quantification

Muscle fibers were incubated with DCA, CA, or vehicle for 72 h. After each treatment, total DNA extraction was performed. The DNA amount was quantified by spectrophotometry. The mitochondrial DNA (mtDNA) was detected and quantified with qPCR using primers to recognize the mitochondrial *Cytb* gene (mt-Cytb, forward: 5′-CCATTCTACGCTCAATCCCCAATA-3′; reverse: 5′-CTACTGGTTGGCCCCCAATTC-3′). The mtDNA levels were normalized with the nuclear gene *B*2*m* (forward: 5′-GGGTCATGGTCTGTGAAGCA-3′; reverse: 5′-CAGAGGCTCTATCGCGGAAA-3′) and detected with qPCR using the SyBR Green method. PCR was performed in triplicate using an Eco Real-Time PCR System (Illumina, San Diego, CA, USA).

### 2.4. Mitochondrial Mass and Morphology

Immunofluorescence for TOM20 and the Mitotracker Green probe (Thermofisher Scientific, Waltham, MA, USA) were used to quantify mitochondrial mass. The Mitotracker Red CMXROS probe (Thermofisher Scientific, Waltham, MA, USA) was used to evaluate morphology. At the end of each treatment, the muscle fibers were washed with Hank’s balanced salt solution (HBSS) and incubated with a Mitotracker Red CMXROS Probe (20 nM) or Mitotracker Green (20 nM) in HBSS 1X for 30 min at 37 °C, 5% CO_2_. Corresponding fibers were washed with HBSS, fixed with paraformaldehyde 4%, and incubated with the anti-TOM20 antibody (1:100, Cell Signaling, Danvers, MA, USA) overnight. AlexaFluor 488 conjugated anti-rabbit (Thermofisher Scientific, Waltham, MA, USA) was used as the secondary antibody. Images were captured using a confocal microscope Leica SP8 and analyzed using ImageJ software (NIH, Bethesda, MD, USA) with the tool Mitochondrial Network Analysis (MiNA).

### 2.5. Mitochondrial Membrane Potential

Muscle fibers were treated with DCA, CA, or vehicle for 72 h. At the end of the experiments, they were incubated with 10 nM Tetramethylrhodamine ethyl ester (TMRE) (Thermofisher Scientific, Waltham, MA, USA) at 37 °C in a 5% CO_2_ incubator for 30 min. Furthermore, images were captured in live fibers using a confocal microscope Leica SP8 and analyzed using the software ImageJ (NIH, Bethesda, MD, USA) with the MiNA tool. Controls with carbonyl cyanide-p-trifluoromethoxyphenylhydrazone (FCCP) (150 nM) and oligomycin (1 μM) were used.

### 2.6. Mitochondrial Reactive Oxygen Species

Muscle fibers were treated with DCA, CA, or vehicle for 72 h. At the end of the experiments, they were incubated with 5 μM Mitosox Red (Thermofisher Scientific, Waltham, MA, USA) at 37 °C in a 5% CO_2_ incubator for 30 min. Mitosox is a fluorogenic dye explicitly targeting mitochondria in live fibers. Its oxidation by superoxide produces red fluorescence. Images were captured using a confocal microscope Leica SP8 and analyzed using ImageJ software with the MiNA tool. Mitosox Red is a probe the entrance of which into mitochondria depends on the mitochondrial membrane potential. For this reason, the fluorescence intensity of Mitosox was normalized with the TMRE signal [18].

### 2.7. Analysis of Cellular Respiration and Mitochondrial Energetics

Muscle fibers were seeded in XF24-3-well microplates (Agilent Technologies, MA, USA) covered with Matrigel. The fibers were incubated with DCA and CA for 72 h. Then, the fibers were set without CO_2_ for 1 h. The XF assay medium was low-buffered bicarbonate-free Dulbecco’s Modified Eagle Medium (DMEM) (pH 7.4) and replicated the glucose and pyruvate/glutamax composition of the respective experimental conditions. Evaluation of the oxygen consumption rate (OCR) was undertaken at different stages: basal respiration, ATP-linked respiration, H^+^-leak, maximal respiratory capacity, spare respiratory capacity, and non-mitochondrial respiration, using modulators of cellular respiration (1 μM oligomycin, 1 μM carbonyl cyanide p-trifluoromethoxyphenylhydrazone (FCCP), rotenone, and antimycin A) as previously described [19,20]. In addition, a Seahorse Bioscience XF24-3 Extracellular Flux Analyzer (Agilent Technologies, MA, USA) was used to evaluate the mitochondrial parameters normalized to protein content/well within the Seahorse plate. For Seahorse XF analyzer studies, data points were collected for each experimental condition from a minimum of three replicates, with each experiment being conducted at least three times. After detection, the cellular protein content was quantitated with a MicroBCA kit, and OCR was normalized accordingly.

### 2.8. Statistical Analysis

The statistical analysis of the data was performed with the Prism 8.0 analysis software (GraphPad Software, San Diego, CA, USA). The normality of the data was determined. Normal data were analyzed with a *t*-test to compare two groups; one- or two-way ANOVA was used as appropriate with a Tukey post hoc test to analyze three or more groups. Differences were considered significant when *p* < 0.05.

## 3. Results

### 3.1. Decreased Mitochondrial Mass Induced by Deoxycholic and Cholic Acids Is Dependent on TGR5 Expression

**Figure 1:**
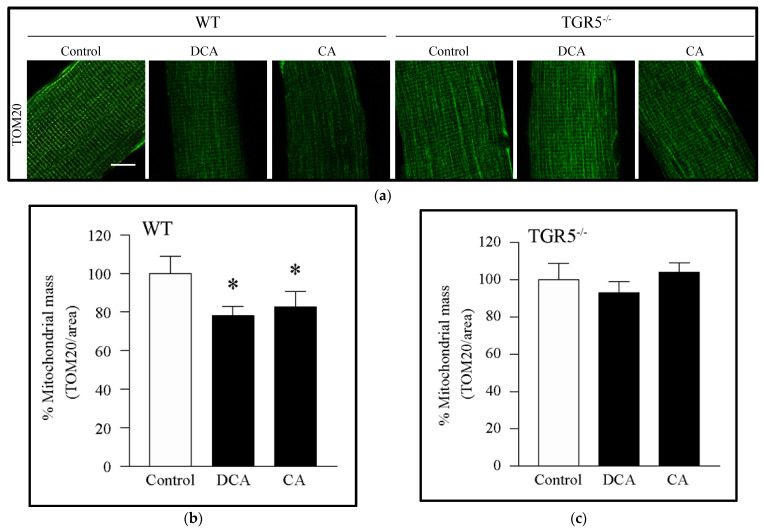
Decreased mitochondrial mass induced by deoxycholic acid (DCA) and cholic acid (CA) in muscle fibers involves a TGR5-dependent mechanism. Extensor digitorum longus (EDL) muscle fibers from WT and TGR5^−/−^ mice were incubated with DCA or CA for 72 h. Then, indirect immunofluorescence for TOM20 was performed. (**a**) Representative images for TOM20. Scale bar = 50 μm. (**b**,**c**) Analysis of TOM20 immunostaining to quantify mitochondrial mass expressed as a percentage in WT (b) or TGR5^−/−^ (**c**) fibers. Values correspond to the means ± SEM (*n* = 3, * *p* < 0.05 vs. control condition; one-way ANOVA and Tukey’s multiple comparison test).

We evaluated the effect of DCA and CA on mitochondrial mass in EDL-isolated muscle fibers using imaging. For this, we performed immunostaining against TOM20 as a mitochondrial mass marker because this is a protein of the outer mitochondrial membrane and can be detected entirely independent of mitochondrial membrane potential. The main advantage of this strategy that it makes it possible to determine the mitochondrial mass independently of the mitochondrial membrane potential, unlike the other methods typically used, such as Mitotracker Red staining, in which access to mitochondria is dependent on the method of mitochondrial membrane potential. Figure 1a,b shows that EDL-isolated fibers from WT mice incubated with DCA and CA decreased the immunoreactivity of TOM20 by 20%. Interestingly, no changes were observed in the TOM20 levels in EDL-isolated fibers from TGR5^−/−^ mice incubated with DCA and CA (Figure 1a,c). To complement this result, we incubated muscle fibers with Mitotracker Green probe, which labels mitochondria independently of mitochondrial potential. The result showed that DCA and CA decreased the mitochondrial mass in EDL fibers isolated from WT mice, which was prevented in fibers from TGR5^−/−^ mice (Supplementary Figure S1a,c, right panel).

We observed that the Mitotracker Red stain changed from a homogenous pattern in WT control fibers (Supplementary Figure S2a, left panel) towards a punctate pattern in WT DCA- and CA-treated fibers (Supplementary Figure S2a, left panel). The quantification of these effects is shown in Supplementary Figure S2b, demonstrating that DCA and CA increased by 4.0 ± 0.3-fold and 2.2 ± 0.3-fold, respectively, in the WT fibers. Interestingly, the changes in the Mitotracker Red pattern induced by bile acids were prevented in EDL fibers isolated from TGR5^−/−^ mice (Supplementary Figure S2a,c).

**Figure 2:**
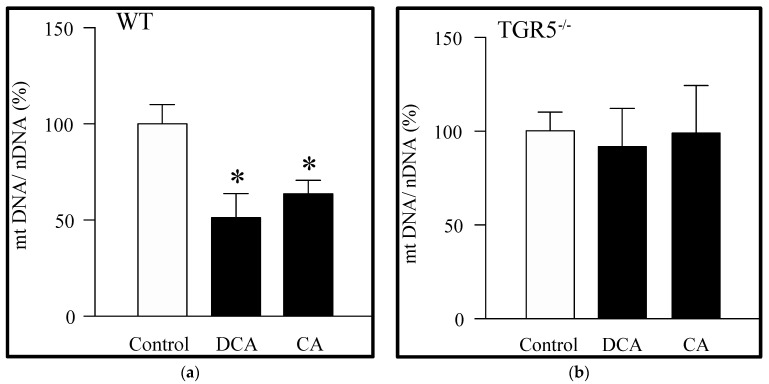
Deoxycholic acid (DCA) and cholic acid (CA) decrease the amount of mitochondrial DNA in muscle fibers through a TGR5-dependent mechanism. Extensor digitorum longus (EDL) muscle fibers from WT and TGR5^−/−^ mice were incubated with DCA or CA for 72 h, and mitochondrial DNA (mtDNA) and nuclear DNA (nDNA) were determined. (**a**) mtDNA detected in WT fibers incubated with DCA or CA. (**b**) mtDNA detected in TGR5^−/−^ fibers incubated with DCA or CA. Values correspond to the mean ± SEM. (*n* = 3, * *p* < 0.05 vs. control condition; one-way ANOVA and Tukey’s multiple comparison test).

We then evaluated the effects of DCA and CA on the amount of mitochondrial DNA (mtDNA). DCA and CA decreased the mtDNA in EDL muscle fibers from WT mice (Figure 2a). However, this phenomenon was abolished in EDL muscle fibers from TGR5^−/−^ mice for both bile acids (Figure 2b). Together, these results suggest that DCA and CA decreased the mitochondrial mass via the TGR5 receptor.

### 3.2. Deoxycholic and Cholic Acids Decreased Mitochondrial Potential and Bioenergetics through a TGR5-Dependent Mechanism

**Figure 3:**
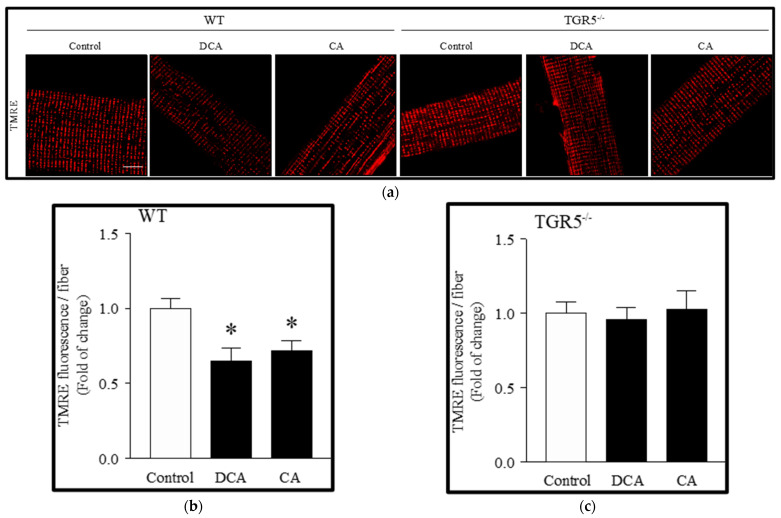
Decreased mitochondrial potential induced by deoxycholic acid (DCA) and cholic acid (CA) in muscle fibers involves a TGR5-dependent mechanism. Extensor digitorum longus (EDL) muscle fibers from WT and TGR5^−/−^ mice were incubated with DCA or CA for 72 h. Then, fibers were incubated with TMRE. (**a**) Representative images for TMRE. Scale bar = 20 μm. (**b**,**c**) Analysis of TMRE stain in WT (**b**) and TGR5^−/−^ (**c**) fibers. Values are expressed as fold of change and correspond to means ± SEM (*n* = 3, * *p* < 0.05 vs. control condition; one-way ANOVA and Tukey’s multiple comparison test).

We determined the effect of DCA and CA on mitochondrial potential with TMRE staining. Figure 3a shows that the TMRE signal decreased after treatment with DCA (35 ± 8.8%) and CA (27 ± 7.4%) in EDL muscle fibers from WT mice, as evidenced in the quantification (Figure 3b). However, similarly to other results, EDL muscle fibers from TGR5^−/−^ mice did not change the TMRE signal observed in the control when incubated with DCA and CA (Figure 3a), as evidenced in the quantitative analysis (Figure 3c).

**Figure 4:**
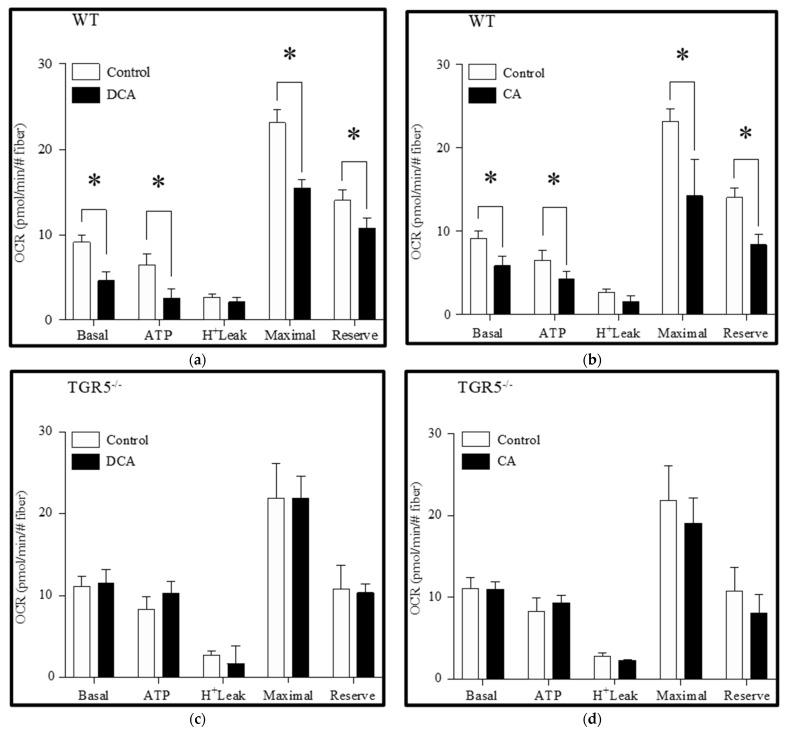
Deoxycholic acid (DCA) and cholic acid (CA) decrease the oxygen consumption rate (OCR) in muscle fiber through a TGR5-dependent mechanism. Skeletal muscle fibers from WT and TGR5^−/−^ mice were incubated with DCA or CA for 72 h, and the basal, ATP-linked, H^+^-leak, maximal, and spare OCR were determined as described in the Materials and Methods section. (a,b) Analysis for OCR in WT fibers incubated with DCA (**a**) or CA (**b**). (**c**,**d**) Analysis for OCR in TGR5^−/−^ fibers incubated with DCA (**c**) or CA (**d**). Values correspond to the mean ± SEM (*n* = 3, * *p* < 0.05, *t*-test).

Furthermore, we determined the effects of the bile acids DCA and CA on the OCR obtained with the Seahorse equipment. For this, measurements were made in different steps and associated with different events: first, the OCR associated with the mitochondrial respiration without stimulus (basal) was determined. Then, oligomycin was added to the medium to inhibit the ATP synthase. ATP synthase inhibition built up the membrane potential and, as a consequence, the respiratory chain was arrested and a decreased OCR was observed. Oligomycin-insensitive OCR was due to a proton leak or uncoupled respiration. The difference between basal and oligomycin-insensitive respiration indicated the OCR required to synthesize ATP (ATP-linked OCR). Immediately after, FCCP was added to collapse the membrane potential and stimulate the maximal OCR. The difference between the maximal and basal OCR reflected the spare respiratory capacity. Finally, antimycin A plus rotenone was added to completely inhibit the ETC and determine the non-mitochondrial respiration. Our results showed that DCA treatment (Figure 4a) decreased the basal OCR (control = 9.16 ± 0.9 vs. DCA = 4.56 ± 1.0), the ATP-linked OCR (control = 6.65 ± 1.1 vs. DCA = 2.47 ± 1.4), the maximal respiration (control = 23.18 ± 1.7 vs. DCA = 15.44 ± 1.1), and the spare OCR (control = 13.91 ± 1.4 vs. DCA = 10.84 ± 1.2) (Figure 4a) in WT fibers. No changes were observed for the oligomycin-insensitive OCR. CA treatment (Figure 4b) showed similar results in WT fibers (basal OCR: control = 9.16 ± 0.9 vs. CA: 5.82 ± 1.2; ATP-linked OCR: control = 6.65 ± 1.1 vs. CA = 4.34 ± 1.0; maximal respiration: control = 23.18 ± 1.7 vs. CA = 14.19 ± 4.1; spare OCR: control = 13.91 ± 1.4 vs. CA = 8.54 ± 1.5). Interestingly, all these effects shown for WT fibers subjected to DCA and CA treatments were abolished in TGR5^−/−^ fibers (Figure 4c,d).

These results suggest the participation of the TGR5 receptor in the bile acid-induced decline in mitochondrial potential and OCR in skeletal muscle fibers.

### 3.3. Diminution of Mitochondrial OXPHOS Complexes Induced by Deoxycholic and Cholic Acids Is Attenuated by TGR5 Deficiency

**Figure 5:**
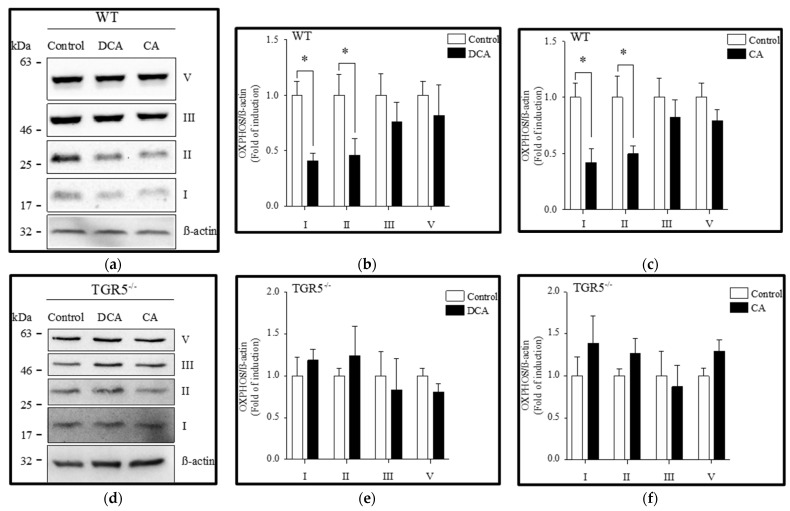
Deoxycholic acid (DCA) and cholic acid (CA) decrease oxidative phosphorylation (OXPHOS) complexes I and II from mitochondria in muscle fiber through a TGR5-dependent mechanism. Skeletal muscle fibers from WT and TGR5^−/−^ mice were incubated with DCA or CA for 72 h. Protein levels of mitochondrial complexes I, II, III, and V were detected by Western blot analysis. (**a**–**c**) Analysis of WT fibers incubated with DCA (**a**,**b**) or CA (**a**,**c**). (**d**–**f**) Analysis of TGR5^−/−^ fibers incubated with DCA (**d**,**e**) or CA (**d**,**f**). Values correspond to the mean ± SEM (*n* = 3, * *p* < 0.05, *t*-test).

We evaluated the effects of DCA and CA on the protein levels of OXPHOS complexes using Western blot analysis. Figure 5a shows that DCA and CA decreased complexes I and II, whereas complexes III and V were unchanged in EDL muscle fibers from WT mice. Furthermore, the quantification indicated that complexes I and II decreased by 0.42 ± 0.07-fold and 0.48 ± 0.14-fold, respectively, after treatment with DCA (Figure 5b). CA reduced the same complexes, I and II, by 0.43 ± 0.12-fold and 0.49 ± 0.09-fold, respectively (Figure 5c). However, EDL muscle fibers from TGR5^−/−^ mice (Figure 5d) did not show changes in any of the mitochondrial OXPHOS complexes after incubation with DCA (Figure 5e) or CA (Figure 5f).

### 3.4. Deoxycholic and Cholic Acids Increased Mitochondrial ROS through a TGR5-Dependent Mechanism

**Figure 6:**
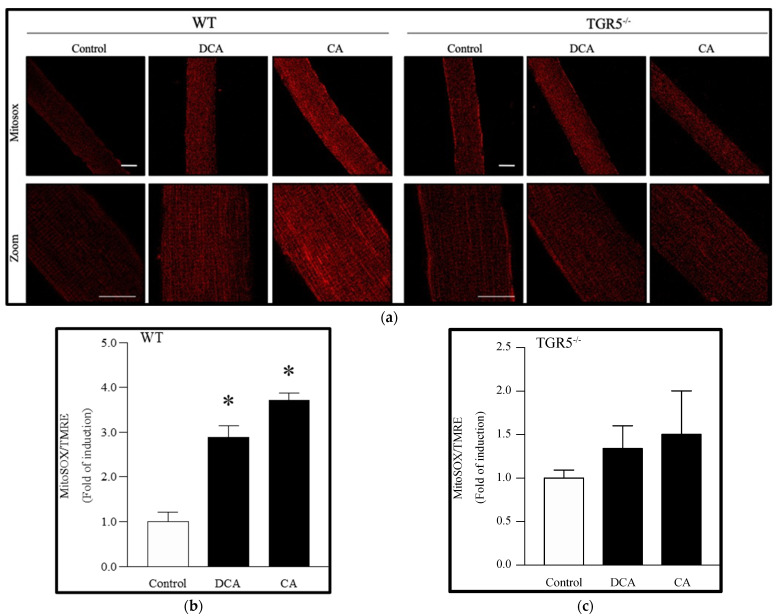
Increased mitochondrial reactive oxygen species (ROS) levels induced by deoxycholic acid (DCA) and cholic acid (CA) in muscle fibers involve a TGR5-dependent mechanism. Skeletal muscle fibers from WT and TGR5^−/−^ mice were incubated with DCA or CA for 72 h. Then, fibers were incubated with a Mitosox probe. (**a**) Representative images for the Mitosox signal (upper panel, lower panel zoom). Scale bar = 20 μm. (**b**,**c**) Analysis of the Mitosox signal normalized by the TMRE signal in WT (**b**) or TGR5^−/−^ (**c**) fibers. Values are expressed as the fold of induction and correspond to the means ± SEM (*n* = 3, * *p* < 0.05 vs. control condition; one-way ANOVA and Tukey’s multiple comparison test).

We evaluated the effect of DCA and CA on mitochondrial ROS by detecting the signal of the Mitosox probe. Figure 6a shows that the Mitosox signal increased after treatment with DCA (2.75 ± 0.07-fold) and CA (3.55 ± 0.07-fold) in EDL muscle fibers from WT mice, as evidenced in the quantification (Figure 6b). However, as expected, no changes were observed in DCA- and CA-treated TGR5^−/−^ fibers compared to controls (Figure 6c).

These results indicate that DCA and CA increase mtROS through a TGR5-dependent mechanism in skeletal muscle fibers.

## 4. Discussion

We demonstrated in the present study that DCA and CA induce mitochondrial dysfunction, as determined by decreased mitochondrial parameters, such as mass, DNA, potential, and OCR, as well as increased mtROS. Importantly, we also described the critical role of TGR5 expression in the harmful effects induced by DCA and CA on mitochondrial functions.

The data from our study showed a homogeneous and highly concentrated distribution of mitochondria in WT muscle fibers (control), consistent with that described in the literature [21,22,23,24,25,26,27]. This fact is consistent with the mitochondrial organization in muscle tissue, in which mitochondria are located between myofibrils with a high degree of packing and limited movement and mobility. Under this condition, it has been reported that fusion and fission events are less detectable, and mitochondrial communication is generated through nanotunnels [28,29,30]. Our results showed that DCA- and CA-treated WT muscle fibers displayed more significant mitochondrial fragmentation and loss of the connection between them, which could be explained by a reduction or elimination of nanotunnels, leaving isolated and localized mitochondria between the myofibrils. In addition, when mitochondrial fragmentation is present, this phenomenon can be associated with the coexistence of depolarized and hyperpolarized mitochondria.

Our results showed that DCA and CA significantly reduced mitochondrial membrane potential. This result was consistent with that described for other models of muscle atrophy, such as immobilization or aging [31,32]. In addition, we showed that DCA and CA alter OCR, inducing a decrease in basal respiration and maximum respiration, consistent with the reduction in mitochondrial biomass observed. The mechanism underlying the decline in the mitochondrial membrane potential caused by DCA and CA in muscle fibers was not evaluated. However, our results suggest that this decrease was not related to mitochondrial uncoupling, since the OCR leak values, typically associated with dissipation of potential that does not generate energy, were unchanged by DCA and CA. This fact contrasts with the results reported for brown adipose tissue, where bile acids increased ETC activity to increase energy expenditure (or heat generation) via TGR5 [15].

A possible explanation for the reduction in the mitochondrial membrane potential induced by DCA and CA is the decreased protein levels of OXPHOS complexes I and II, which are initiators of electron transport and generate proton transport from the mitochondrial matrix to the intermembrane space. Additionally, complex I also acts as a proton pump, contributing to this potential difference. The decrease in complexes I and II levels could have altered the OXPHOS function, which would explain the decline in the potential and oxygen consumption rate. Furthermore, our results showed that DCA and CA reduced the spare mitochondrial capacity, which would explain the lower capacity to satisfy the energy demand shown by these cells. These findings are consistent with studies related to increased bile acids and mitochondrial dysfunction [12,14,33,34,35]. Concomitantly with other explanations, DCA and CA could cause the dissipation of the potential through the opening of the mitochondrial permeability transition pore (mPTP), as has been demonstrated in previous studies with hepatic mitochondria exposed to hydrophobic bile acids [36,37]. These results showed the loss of the electrochemical gradient across the inner membrane, uncoupling of oxidative phosphorylation, and colloid-osmotic swelling of mitochondria, events that result in the onset of cellular necrosis and may also be central to the apoptotic process [38,39,40], which could explain some of our previously obtained results [9,41]. From another perspective, this potential dissipation could also depend on chloride channels (CLIC) located in the mitochondrial membrane, the expression of which is regulated by apoptotic factors [42,43]. The CLIC overexpression results in the induction of several apoptotic events [43].

This study also showed an increase in the mtROS levels induced by DCA and CA, which could have been the consequence of a diminution of the antioxidant system in the mitochondria and a higher ROS production derived from alterations in the OXPHOS function. This increase in mtROS can generate oxidative damage in the OXPHOS components and, in this way, contribute to the development of mitochondrial dysfunction [31]. Increased mtROS occur in the mitochondrial matrix, where mtDNA resides. Mitochondrial ROS generate mtDNA mutations and deletions affecting the mtDNA copy number and the expression and quality of mtDNA-encoded ETC proteins, causing a vicious cycle producing more ROS. Consequently, the repressor of mitochondrial biogenesis, PARIS, accumulates in the cytosol and blocks PGC-1α, the master regulator of mitochondrial biogenesis [44,45]. This metabolic axis may explain the 50% reduction in mtDNA content when muscle fibers were exposed to DCA and CA. Final mtDNA content is a balance between mtDNA biogenesis (replication) and degradation by the DNA polymerase POLγ, as well as by mitophagy. On the other hand, mitochondrial biomass, measured as mitochondrial area (surface) by the Mitotracker probe, is the balance between membrane biogenesis, which occurs in tight association with the endoplasmic reticulum, and degradation, which is dependent on phospholipases and mitophagy [45]. Thus, both processes, mtDNA and membrane biogenesis, can be regulated independently, which supports our observation of a 20% reduction in mitochondrial biomass when muscle fibers were exposed to DCA and CA.

Mitochondria are bioenergetic modulators in the skeletal muscle fibers and play a crucial role in muscle physiopathology and metabolism. Under conditions of muscle atrophy or sarcopenia, it has been found that dysregulation of the biogenesis/mitophagy processes occurs, generating a loss of mitochondrial content and accumulation of dysfunctional mitochondria. Dysfunctional mitochondria in muscle fibers can regulate various mechanisms linked to muscle atrophy; mainly, those related to degradation pathways [46,47]. From this perspective, increased mtROS could produce an increment of myonuclear apoptosis by activating the intrinsic apoptotic pathway, which is dependent on the mPTP/cytochrome c pathway, activating cascades of caspase signaling and leading to nuclear DNA fragmentation [3,31]. These possible mechanisms are in agreement with our previous studies on sarcopenia induced by CLD. We demonstrated that a murine model of CLD presents high levels of bile acids together with muscle weakness, muscle atrophy, and myonuclear apoptosis [9,41]. In addition, a similar effect has been observed with high concentrations of bile acids in hepatocytes, showing a cytotoxic effect mediated by the opening of mPTP, suggesting a central role as a marker of apoptosis [14,33]. On the other hand, an increase in mtROS can generate oxidative stress at the cytoplasmic level, causing oxidative damage in protein components or other organelles and damage to cell membranes. Moreover, as previously mentioned, the increase in ROS produces NF-κB activation; this is associated with the increase in MuRF-1 expression that we have previously reported, which could potentiate the decrease in the myofibrillar protein content observed in skeletal muscle cells exposed to DCA and CA [48].

The data presented in our analysis support the hypothesis that mitochondrial dysfunction induced by DCA and CA is dependent on TGR5 expression. These results agree with our previous reports, which demonstrated that these bile acids, through a TGR5-dependent mechanism, induce muscle wasting by activating the ubiquitin-proteasome system, myonuclear apoptosis, and oxidative stress [10]. In addition, our results complement our previous report on TGR5-dependent sarcopenia in a murine model of CLD, in which muscle weakness, atrophy, and exacerbated protein degradation were developed [7,41]. Thus, our present results suggest that mitochondrial dysfunction could contribute to the sarcopenic phenotype in this model associated with minor ATP production, low muscle strength, development of oxidative stress, and increased proteostasis [41]. In addition, our results are in concordance with previous studies that demonstrated TGR5-dependent mitochondrial dysfunction in tissues other than muscle [49,50].

The mechanism by which the DCA/CA-TGR5 axis induces mitochondrial dysfunction in skeletal muscle is unknown. In other tissues, such as brown adipose tissue, bile acids induce mitochondrial uncoupling via TGR5 [15]. In this tissue, the increased mitochondrial uncoupling caused by bile acids is due to an increment in UCP1 levels; this leads to the dissipation of energy as heat, which increases energy expenditure by this organelle [15,51]. Although skeletal muscle expresses UCP3 as a protein similarly to the mitochondrial uncoupling protein UCP1, its function has not been clearly described. Studies on the role of UCP3 are controversial, indicating that it has different functions from UCP1 [52,53,54]. Thus, further studies must be performed to determine the mechanism that links the TGR5 receptor with mitochondrial function in skeletal muscle.

## 5. Conclusions

This study demonstrated that CA and DCA induce TGR5-dependent mitochondrial dysfunction in muscle fibers. The results show that DCA and CA decreased TOM20 levels, Mitotracker red reactivity, and mitochondrial DNA as parameters of mitochondrial biomass in muscle fibers. We also observed reduced mitochondrial potential, oxygen consumption rate (basal, maximal, spare, and ATP-associated), and OXPHOS complex I and II levels, suggesting decreased mitochondrial capacity in ATP synthesis. In addition, we showed that DCA and CA induced increased mitochondrial ROS (mtROS) production. All these features induced by DCA and CA were dependent on TGR5 expression in skeletal muscle fibers. Additional studies are required to determine the direct mechanism activated by these bile acids that produces all the effects at the mitochondrial level in muscle cells.

## Data Availability

The data presented in this study are available in the article and supplementary material.

## Supplementary Materials

The following supporting information can be downloaded at: https://www.mdpi.com/article/10.3390/antiox11091706/s1. Figure S1: Deoxycholic acid (DCA) and cholic acid (CA) decreased the mitochondrial mass in muscle fiber through a TGR5-dependent mechanism. Skeletal muscle fibers from WT and TGR5^−/−^ mice were incubated with DCA or CA for 72 h. Further, fibers were incubated with a Mitotracker Green probe for 30 min. (a) Representative images for the Mitotracker Green signal. (b,c) Analysis of intensity of fluorescence in muscle fibers from WT (b) or TGR5^−/−^ (c) mice. Values are expressed as fold of induction and correspond to the means ± SEM (*n = 3*, * *p* < 0.05 vs. control condition; one-way ANOVA and Tukey’s multiple comparison test). Figure S2: The mitochondrial pattern was altered by deoxycholic acid (DCA) and cholic acid (CA) in muscle fiber through a TGR5-dependent mechanism. Skeletal muscle fibers from WT and TGR5^−/−^ mice were incubated with DCA or CA for 72 h. Further, fibers were incubated with a Mitotracker Red probe for 30 min. (a) Representative images for the Mitotracker Red signal. (b,c) Analysis of the Mitotracker Red-based punctate pattern in muscle fibers from WT (b) or TGR5^−/−^ (c) mice. Values are expressed as fold of induction and correspond to the means ± SEM (*n = 3*, * *p* < 0.05 vs. control condi-tion; one-way ANOVA and Tukey’s multiple comparison test).
