# Supplementary figures and images for: Bile Acids Induce Alterations in Mitochondrial Function in Skeletal Muscle Fibers
Source: Antioxidants (Basel). 2022 Aug 30;11(9):1706. doi: 10.3390/antiox11091706 (PMC9495846; doi:10.3390/antiox11091706)

**a**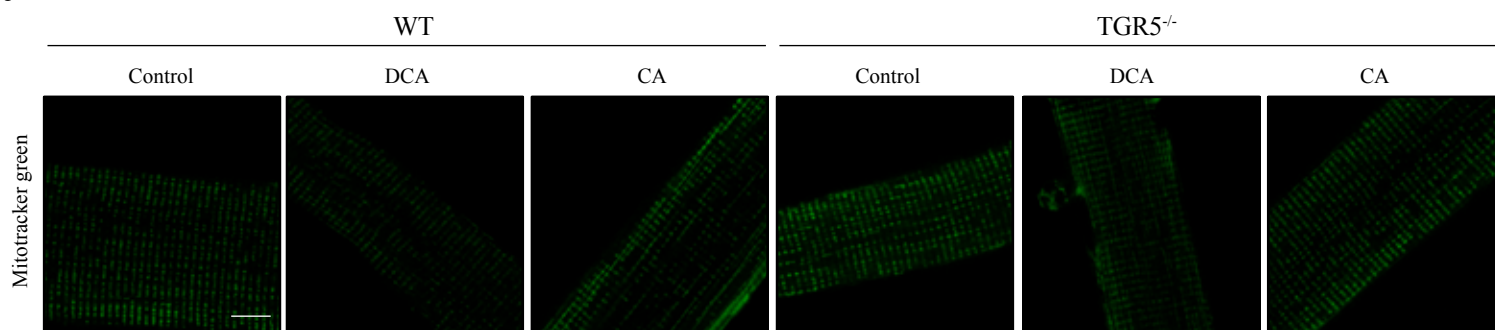**b**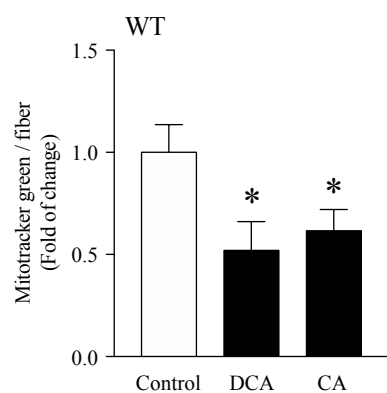**c**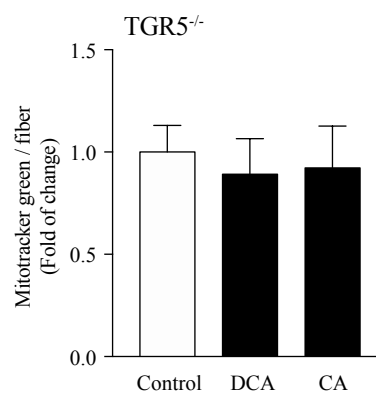

**Supplemental Figure S1**

**a**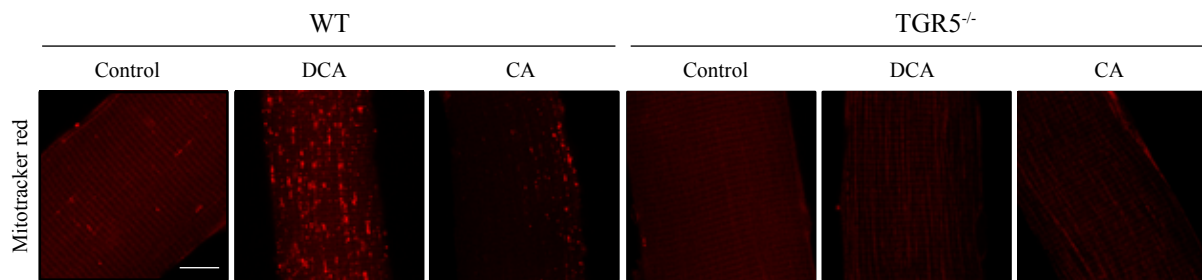**b**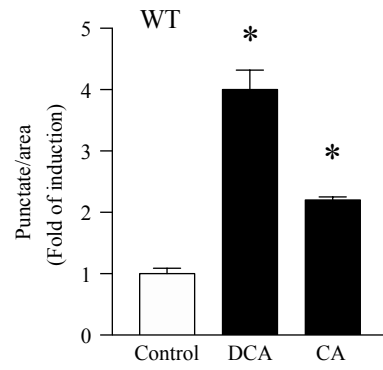**c**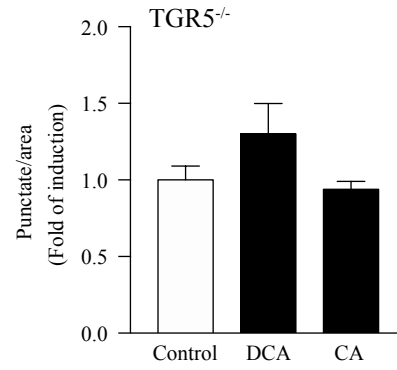

# Supplemental Figure S2

Supplement: Supplementary file 1 [file antioxidants-11-01706-s001.zip › antioxidants-1847652-supplementary.pdf]
